# Rectal cancer presentation during the COVID-19 pandemic: Are decreasing screening rates leading to an increase in acute presentations?

**DOI:** 10.1371/journal.pone.0291447

**Published:** 2023-09-14

**Authors:** Tia S. Sutton, Scarlett Hao, Mizuki Suzuki, Aimei Chua, Anna Lisa Ciarrocca, Michael D. Honaker

**Affiliations:** 1 Department of Surgery, East Carolina University Brody School of Medicine, Greenville, NC, United States of America; 2 East Carolina University Brody School of Medicine, Greenville, NC, United States of America; 3 Division of Surgical Oncology, Department of Surgery, East Carolina University Brody School of Medicine, Greenville, NC, United States of America; Brazilian national cancer institute, BRAZIL

## Abstract

Nearly 23 million adults ages 50–75 are overdue for colorectal cancer (CRC) screening. In March 2020, the Centers for Medicare & Medicaid issued guidance that all non-urgent procedures be delayed due to the COVID-19 pandemic. Screening delays may have effects on the presentation of rectal cancer and the natural history of the disease. The aim of this study was to determine if procedural suspension due to the COVID-19 pandemic was associated with an increased proportion of acute presentations or more advanced stage at diagnosis for patients with rectal cancer. We conducted a single-center, retrospective review of adult patients with new or recurrent rectal adenocarcinoma from 2016–2021. We compared patients presenting before (pre-COVID) to those diagnosed after (COVID) March 1, 2020. Of 208 patients diagnosed with rectal cancer, 163 were diagnosed pre-COVID and 45 patients in the COVID group. Cohorts did not differ among age, sex, race, insurance status, marital status, rurality, or BMI. There was no difference in stage at presentation with the majority diagnosed with stage III disease (40.0% vs 33.3%, p = 0.26). Similar proportions of patients presented acutely (67.5% vs 64.4%, p = 0.71). Presenting symptoms were also similar between cohorts. On adjusted analysis, male sex, white race, and uninsured status were found to have significant impact acuity of presentation, while diagnosis before or after the onset of the pandemic remained non-significant (OR 1.25, 95% CI0.57–2.72; p = 0.59). While screening rates have decreased during the COVID pandemic, patients with rectal cancer did not appear to have an increased level of acuity or stage at presentation. These findings could result from the indolent nature of the disease and may change as the pandemic progresses.

## Introduction

Colorectal cancer (CRC) remains the second leading cause of cancer-related deaths in the United States in both males and females, with nearly 149,500 new cases and 53,000 CRC-related deaths during 2021 alone [1]. It is well-established that screening colonoscopies improve CRC-related mortality. There is up to a 75% reduction in risk of death for patients with left-sided colon cancer, which is attributed to detection and diagnosis at an earlier stage [2, 3]. Previous studies have suggested that even a single colonoscopy is associated with a reduction in long-term risk of CRC of greater than 20 years [4]. Despite CRC screening measures significantly impacting early detection and stage-related survival, nearly 23 million adults ages 50–70 in the United States are overdue for CRC screening [5]. This number may be even larger in the setting of increasing CRC diagnoses in younger patients with subsequent changes in recommendations for earlier and more frequent screening in certain higher risk subpopulations [6].

In March 2020, the Centers for Medicare and Medicaid Services (CMS) issued guidance that all non-urgent procedures be delayed due to the COVID-19 pandemic [7]. In addition to a 49–61% decline in adult primary care and gastroenterology visits during this time, there were also specific challenges with early COVID-related endoscopic disinfection practices, adding to barriers for patients to receive screening colonoscopies [5]. This led to a greater than 90% relative reduction in colonoscopies performed in both the U.S. and England during April 2020, with deficits in subsequent CRC diagnosis and treatment initiation persisting up to 6 months in duration [8].

As CRC screening is central to prevention and early detection, the consequences of delayed screening may potentially result in more acute presentations and with more advanced stages at diagnosis [8]. The primary aim of this study was to determine if there was a change in the acuity of presentation and stage at diagnosis for patients with rectal adenocarcinoma as these patients are more likely to present symptomatically and have a stronger association with endoscopic screening [4, 9], following the COVID-19 related procedural suspension.

## Methods

### Data source and patient selection

A single-institution, retrospective cohort study was conducted on all patients **≥** 18 years old diagnosed with rectal adenocarcinoma between January 1, 2016 and December 12, 2021. The institution is a tertiary care center and a Commission on Cancer-accredited comprehensive cancer center. The institutional tumor registry was queried for patients meeting inclusion criteria. Variables and outcomes of interest were collected utilizing the electronic medical record. Patients with pathology other than adenocarcinoma were excluded. Rectal cancer was defined anatomically as tumors occurring in the upper, mid, and lower rectum by endoscopy as measured 15 cm from the anal verge or receiving therapy consistent with a diagnosis of rectal cancer (e.g., neo-adjuvant chemotherapy and/or radiation). This study was approved by the University and Medical Center Institutional Review Board (UMCIRB 21–000732) and deemed except, therefore, informed consent waived given the retrospective nature of the study. Data was de-identified after the collection process.

Clinical characteristics examined were date of diagnosis, chief complaint at presentation, acuity of presentation, body mass index (BMI), stage (as determined by the American Joint Committee on Cancer 7^th^ and 8^th^ edition based upon date of diagnosis), and Charlson Comorbidity Index (CCI) [10–12]. Screening was defined as a patient meeting criteria for colorectal cancer screening and without any symptoms suggestive of a malignant process. Acuity was determined by the setting of presentation; acute presentation was defined as those presenting to either the emergency department or already admitted to the hospital for rectal cancer related symptoms, and non-acute presentation was defined as those evaluated in the outpatient clinic setting. The patient’s county of residence was defined as rural or metropolitan/urban according to the Federal Office of Rural Health Policy [13]. Patients were categorized as pre-COVID if they had a rectal cancer diagnosis before March 1, 2020 and COVID if they presented after this date.

### Statistical analysis

Descriptive statistics are presented as the number of non-missing observations and percentage for each category per cohort for categorical variables and by mean and standard deviation or median and interquartile range for continuous variables. Significance was tested using chi-square or Fischer’s exact for categorical variables and a two tailed t-test or Wilcoxon rank sum test for continuous variables, as appropriate. Our primary outcomes were stage at diagnosis, acuity of presentation, and chief complaint at presentation. Given the discrepancy in stage II disease in the two cohorts, a Cochran-Armitage test for trends was performed. To adjust for confounding variables and to evaluate the association of covariates, we conducted a multivariable, binary logistic regression for the primary outcome of presentation acuity. Covariates were selected *a priori* and included age, gender, COVID group, race, payor status, rurality, and marital status. Tests of significance were two-sided, and a p-value < 0.05 was considered significant. Analyses were performed with SPSS statistical software (version 28, IBM Corp, Armonk, NY).

## Results

### Patient characteristics

A total of 208 patients met inclusion criteria; 163 patients comprised the pre-COVID cohort, and 45 in the COVID cohort. The cohorts were not statistically different in demographic characteristics (Table 1). 63.9% of the patients were male and 62.0% were white. The mean age at diagnosis was 62 years old in the pre-COVID group and 61 years old in the COVID group. The cohorts were similar with respect to payor status, and both were mainly Medicare-insured (44.2% pre-COVID, 40.0% COVID). The cohorts also did not differ in comorbidity burden, as indicated by the Charlson-Deyo Comorbidity Index (2 [2–4] vs 2 [2–3], p = 0.61).

**Table 1:** Patient demographics.

Variable	Category	Pre-COVID (n = 163)	COVID (n = 45)	p-value
Age^✢^		62.2 (±12.1)	60.5 (±12.4)	0.21
Gender	Female	55 (33.7%)	20 (44.4%)	0.19
	Male	108 (66.3%)	25 (55.6%)	
Race/Ethnicity	White	102 (62.6%)	27 (60.0%)	0.80
	Black	55 (33.7%)	17 (37.8%)	
	Other	6 (3.7%)	1 (2.2%)	
Insurance	Private	58 (35.6%)	18 (40.0%)	0.51
	Medicare	72 (44.2%)	18 (40.0%)	
	Medicaid	26 (16.0%)	5 (11.1%)	
	Uninsured	7 (4.3%)	4 (8.9%)	
Marital Status	Single	31(19.0%)	11 (25.0%)	0.73
	Married	79 (48.5%)	21 (47.7%)	
	Widowed	21 (12.9%)	6 (13.6%)	
	Divorced/Separated	32 (19.6%)	6 (13.6%)	
BMI^†^		27.0 [22.2–32.0]	27.6 [23.0–33.0]	0.27
Rurality	Rural	111 (68.1%)	34 (75.6%)	0.34
	Urban	52 (31.9%)	11 (24.4%)	
Charlson Comorbidity Index^†^		3 [2–4]	3 [2–3]	0.61

✢Reported as mean (±standard deviation)

†Reported as median [IQR]

### Primary outcomes

Stage at presentation was similar between cohorts. The majority of patients presented with stage III disease (40.0% pre-COVID, 33.3% COVID; p = 0.26) and in an acute setting (67.5% pre-COVID, 64.4% COVID; p = 0.71) (Tables 2 and 3). In regard to stage, there was found to be no trend between cohorts and stage (p = 0.330). Bleeding was the most common presenting symptom (50.3% pre-COVID, 42.2% COVID), and only 24.5% of patients pre-COVID and 24.4% COVID presented after undergoing CRC screening. There was no statistical difference in the presenting symptom between cohorts (p = 0.65, Table 3).

**Table 2:** Stage at diagnosis.

Variable	Category	Pre-COVID (n = 163)	COVID (n = 45)	p-value
Stage	I	24 (15.0%)	6 (13.3%)	0.26
	II	31 (19.4%)	15 (33.3%)	
	III	64 (40.0%)	15 (33.3%)	
	IV	41 (25.6%)	9 (20.0%)	

**Table 3:** Presentation acuity & symptomatology.

Variable	Category	Pre-COVID (n = 163)	COVID (n = 45)	p-value
Acuity of Presentation	Acute	110 (67.5%)	29 (64.4%)	0.71
	Non-acute	53 (32.5%)	16 (35.6%)	
Presentation	Screening	40 (24.5%)	11 (24.4%)	0.65
	Bleeding	82 (50.3%)	19 (42.2%)	
	Pain	17 (10.4%)	7 (15.6%)	
	Obstruction	7 (4.3%)	1 (2.2%)	
	Other	17 (10.4%)	7 (15.6%)	

After adjusting for confounding variables (age, gender, COVID group, race, rurality, insurance status, and marital status), there remained no difference in the primary outcome of acuity of presentation between the pre-COVID and COVID cohorts (OR 1.24, 95% CI 0.57–2.72; p = 0.59). However, male gender (OR 2.74, 95% CI 1.30–5.80; p = 0.008), no payor status (OR 4.67, 95% CI 1.10–19.82; p = 0.037), and white race (OR 2.47, 95% CI 1.21–5.04; p = 0.01) were significantly associated with an increase in acuity of presentation (Table 4).

**Table 4:** Adjusted multivariate analysis for primary outcome of acuity.

Variable		Odds Ratio	95% Confidence Interval	p-value
Age		0.99	0.96–1.02	0.62
Gender	Female	Reference		
	Male	2.74	1.30–5.80	0.008
COVID	Pre-COVID	Reference		
	COVID	1.24	0.57–2.72	0.59
Race	Black	Reference		
	White	2.47	1.21–5.04	0.01
	Other	0.44	0.04–4.47	0.49
Rurality	Rural	Reference		
	Urban	0.93	0.45–1.88	0.85
Insurance	Private	Reference		
	Medicare	1.79	0.79–4.09	0.17
	Medicaid	1.62	0.59–4.48	0.35
	Uninsured	4.67	1.10–19.82	0.037
Marital Status	Divorced/Separated	Reference		
	Single	0.36	0.18–1.00	0.05
	Married	0.48	0.21–1.12	0.09
	Widowed	1.40	0.44–4.44	0.57

## Discussion

In this single institution, retrospective study of patients diagnosed with rectal adenocarcinoma, we did not find a significant increase in acuity of presentation or more advanced stage at diagnosis among patients diagnosed after the March 2020 COVID-related procedural suspension. However, male gender, white race, and uninsured status were independently associated with an increased likelihood of presenting acutely.

In the U.S., CRC screening rates dropped by 85% over a three month period at the onset of the pandemic, which remained 36% lower than pre-COVID levels by June 2020, equating to 95,000 missed screenings within this time frame alone [14]. In addition to procedural suspensions, patients were urged to reschedule non-urgent outpatient appointments and advised to avoid unnecessary hospital or emergency department visits [7]. As cancer screenings play a pivotal role in the prevention and earlier detection of disease with subsequent mortality reduction, the procedural suspension at the start of the COVID pandemic and resulting decreases and delays in CRC screening rates may result in an increased proportion of patients presenting acutely with rectal adenocarcinoma. Consequently, this could lead to diagnosis at more advanced stages, reducing the mortality benefit of screening by as much as 20% [15]. Delaying CRC screening by 12 months leads to a reduction in the total years of life gained from screening by nearly 10% [15]. Additionally, the lifetime cost of CRC management increases significantly with more advanced stages at diagnosis, with higher treatment costs negating the cost-saving benefits of disease prevention [14, 16]. In contrast to our findings, other studies have demonstrated an increase in emergent presentation after the onset of the COVID pandemic. A retrospective, single institution study by Shinkwin and colleagues found a two-fold increase in patients presenting with large bowel obstruction and an increase in the number of emergency operations for CRC in 2020 compared to 2018–2019 [17] However, patients with both colon and rectal cancer were included and may account for the differences in findings from our study.

During the pandemic, the U.S. also saw a significant rise in unemployment rates, putting 26.6 million individuals and their dependents at risk of losing employer-based insurance [18]. Increased financial strain coupled with the overall psychological stress of the pandemic exacerbates barriers to healthcare that may disproportionately affect already underserved populations [18]. An observational, cross-sectional comparative analysis by Aguiar and colleagues demonstrated a decrease in outpatient referrals and an increased proportion of patients without insurance that presented with newly diagnosed CRC during the interval of March 2020 to July 2020 [19]. Both studies by Shinkwin and Aguiar also found significant increases in patients presenting with T4 cancers and locally advanced disease, respectively [17, 19]. In contrast, the current study did not find presentation at more advanced stages or greater proportions of uninsured patients diagnosed with rectal cancer after the onset of the pandemic. However, after adjusting for confounding variables, we did observe a difference in presentation acuity for patients without insurance.

Patients with lack of insurance, lower income, and living in medically underserved areas are less likely to undergo cancer screening in general [18]. Not only is incidence of CRC 42% and 31% higher among those with lower education status and low socioeconomic neighborhoods, respectively, but there is also higher CRC-related mortality among rural populations [20]. Significant racial disparities exist as well, with black patients having the highest incidence of CRC and CRC-related deaths [21]. These social determinants of health have been shown to increase the severity of presentation of certain illnesses and are likely worsened by the COVID pandemic [18]. Competing health care needs in patients with already strained resources further diminishes patient motivation and ability to seek screening services, which is demonstrated by patients without insurance having a higher likelihood of presenting in the acute setting compared to those with a payor source.

Some of the disparate findings across studies could be attributed to geographic differences in the extent of delay; some locations were able to start screening again relatively quickly, some never stopped, and some experienced prolonged suspensions [22–24]. The institution utilized in this study reopened endoscopy procedures after two months of the CMS-issued procedural suspension, which may have contributed to the lack of changes seen in the current study. A meta-analysis in Italy found that delays of up to 4–6 months did not significantly reduce the benefits of screening, but shifts towards more advanced stages at diagnosis became apparent with delays of 7–12 months, and delays past 12 months was associated with increased mortality rates [25]. However, even with short-term suspensions, the backlog of screening delays continues to carryover. This may have long-term negative consequences that have not yet been observed, especially considering the relatively indolent natural history of this disease process.

The findings of this study must be viewed in light of its limitations. The data obtained is from a single institution, which may limit the generalizability of findings, in addition to the inherent bias, specifically selection bias and observer bias, in its retrospective design. We elected to keep our analyses isolated to rectal cancer given that it is a distinct entity from colon cancer in terms of its work-up, presentation, and treatment [26]. However, this study is also limited by the relatively low number of patients in the COVID cohort. Continued examination is also needed as the pandemic progresses to assess ongoing effects on the diagnosis, management, and outcomes of CRC in light of the changing healthcare landscape resulting from the pandemic. This has the potential to have a significant impact on CRC screening and outcomes. Additionally, the lack of a difference between the cohorts may result from the indolent nature of the disease process and may change with continued evaluation.

## Conclusions

With the suspension of endoscopic procedures due to the COVID-19 pandemic, we did not find a significant change in acuity of presentation or stage at diagnosis for patients with rectal adenocarcinoma. The ongoing pandemic continues to create barriers to adequate care for cancer patients. With each surge of the pandemic there are increasing numbers of patients needing CRC screening and increasing delays. Given the more indolent nature of CRC, changes in screening and subsequent effects on presentation and stage at diagnosis should continue to be evaluated as the pandemic progresses to address potentially poorer outcomes associated with such screening delays.
